# Thrombomodulin Influences the Survival of Patients with Non-Metastatic Colorectal Cancer through Epithelial-To-Mesenchymal Transition (EMT)

**DOI:** 10.1371/journal.pone.0160550

**Published:** 2016-08-11

**Authors:** Yu-Jia Chang, Ya-Wen Cheng, Ruo-Kai Lin, Chi-Chou Huang, William Tzu-Liang Chen, Tao-Wei Ke, Po-Li Wei

**Affiliations:** 1 Graduate Institute of Clinical Medicine, College of Medicine, Taipei Medical University, Taipei, Taiwan, ROC; 2 Graduate Institute of Cancer Biology and Drug Discovery, College of Medical Science and Technology,Taipei Medical University, Taipei, Taiwan, ROC; 3 Graduate Institute of Pharmacognosy, Taipei Medical University, Taipei, Taiwan, ROC; 4 School of Medicine, Chung Shan Medical University, Taichung, Taiwan, ROC; 5 Department of Surgery, Chung Shan Medical University, Taichung, Taiwan, ROC; 6 Division of Colorectal Surgery, Department of Surgery, China Medical University Hospital, Taichung, Taiwan, ROC; 7 Institute of Medicine, Chung-Shan Medical University, Taichung, Taiwan, ROC; 8 Department of Surgery, College of Medicine, Taipei Medical University, Taipei, Taiwan, ROC; 9 Division of General Surgery, Department of Surgery, Taipei Medical University Hospital, Taipei Medical University, Taipei, Taiwan, ROC; 10 Cancer Research Center, Taipei Medical University Hospital, Taipei Medical University, Taipei, Taiwan, ROC; Hunter College, UNITED STATES

## Abstract

**Background:**

Treatment resistance and metastasis are the major causes of death among patients with colorectal cancer (CRC). Approximately 20% of surgically treated patients ultimately develop metastases during the follow-up period. Currently, the TNM system is the only available prognostic test. Therefore, the identification of new markers for CRC remains important. Thrombomodulin (TM), a glycoprotein, is involved in angiogenesis and has been linked to many malignant diseases. However, the function of TM in CRC remains unclear.

**Methods:**

A total of 170 patients with CRC participated in this study. TM expression was analyzed via immunohistochemistry. Univariate (Kaplan-Meier) analysis was used to analyze patient outcomes, including overall survival (OS) and disease-free survival (DFS). TM expression was manipulated using shRNA or an overexpression system. Transwell migration assays, wound healing migration assays, and the xCELLigence biosensor system were used to detect cell proliferative and migratory capacities.

**Results:**

TM expression in the tumor tissues significantly and positively correlated with the DFS and OS of non-metastatic patients with CRC (ps = 0.036 and 0.0218, respectively). Suppression of TM expression increased the proliferation and migration of DLD-1 cells. TM overexpression reduced the cells’ proliferative and migratory capacities. Cyclooxygenase (COX)-2 expression was up-regulated following TM silencing. Furthermore, the association between the migration of colon cancer cells and the levels of TM and epithelial-to-mesenchymal transition (EMT) markers (fibronectin, vimentin and ezrin) was confirmed in HT29 and DLD-1 cells.

**Conclusions:**

Our study demonstrates that patients with non-metastatic CRC display low TM expression in their tumors and exhibit reduced DFS and OS. The enhanced expression of mesenchymal markers and COX-2 may be involved in the mechanisms that underlie recurrence in patients with cancer displaying low TM expression.

## Introduction

Colorectal cancer (CRC) is a leading cause of cancer-related deaths in developed countries [1]. The majority of patients with CRC can be treated surgically; however, approximately 20% of surgically treated patients ultimately develop metastases over the follow-up period [2]. Treatment resistance and metastasis remain the major problems in the management of CRC and the major causes of death in patients with CRC. Epithelial-to-mesenchymal transition (EMT) is a well-known pathological event in cancer metastasis. Metastasis is the migration of individual cells that detach from a primary site, travel through the circulation and seed in distant organs [3]. The molecular changes associated with EMT include the decreased expression of E-cadherin and the increased expression of mesenchymal markers [4,5]. Furthermore, studies have demonstrated that acquired treatment resistance in cancer cells is associated with the up-regulation of EMT in patients with CRC [6,7].

Thrombomodulin (TM), a glycoprotein first identified on the endothelial cell surface, plays an important role in the regulation of blood coagulation and anti-coagulation [8–10]. TM is widely distributed in epithelial cells, keratinocytes, synovial lining cells, and meningeal cells [11–13]. Although TM has not been detected in normal cuboidal, simple columnar, or pseudostratified columnar epithelium [11], TM was detected in adenocarcinomas and associated with prognosis [14,15]. TM was also detected in patients with CRC [16,17], and patients with high TM expression levels had better prognoses [16].

Our previous study demonstrated that the suppression of TM decreases E-cadherin expression and enhances migration in hepatocellular carcinoma (HCCs) [18]. However, the relationship between TM and EMT and the mechanism through which TM influences survival among patients with CRC are elusive. Furthermore, no TM expression pattern has been described among Asian patients with CRC. Here, we sought to determine the TM expression pattern of Asian patients with CRC and to examine whether TM modulates cancer progression and metastasis. We believe that the information described here could help determine whether TM could be used as a biomarker for CRC.

## Methods

### Patients and specimens

CRC tumor tissues were collected from 170 patients (stage 1–3) who underwent curative surgical resection at the Department of Surgery of Taipei Medical University Hospital between December 2008 and December 2010. None of the participants reported a previous history of cancer. The clinical stages and pathological characteristics of the primary tumors were defined according to the criteria of the American Joint Commission on Cancer. Patients with distant metastasis were excluded, and those with nodal involvement received post-operative chemotherapy. Postoperative follow-up visits were scheduled for 1 and 2 months after surgery, every 3 months over the first 2 years, and every 6 months thereafter (or more frequently if necessary). The median follow-up duration after curative resection was 4.8 years.

### Ethics statement

This project was approved by the Institutional Review Board of Taipei Medical University.

Written informed consent was obtained from all participants or guardians prior to the use of their resected specimens.

### Immunohistochemistry (IHC)

Formalin-fixed and paraffin-embedded tissues were sectioned at a thickness of 3 μm. All of the sections were deparaffinized in xylene, sequentially rehydrated using serial dilutions of alcohol, and washed in phosphate-buffered saline. The sections used for TM detection were immersed in citrate buffer (pH 6.0) and heated in a microwave oven twice for 5 min. The primary antibody was a mouse anti-TM monoclonal antibody (1:200 dilution; Santa Cruz, CA, USA). The detailed protocol was described in our previous report [19,20]. Normal colorectal tissue was used as a positive control. Negative controls that did not include the primary antibodies were also prepared. Three observers independently evaluated the results, which were scored for the percentage of positive expression. TM protein was expressed only on the membrane. Anti-TM antibody staining of the cell membrane was considered positive immunostaining, and a lack of anti-TM antibody staining was classified as negative immunostaining. We counted the number of TM-positive cells in colorectal tumor tissues from three independent fields using a high-power field (objective lens 40×). Cells that were positively stained for the anti-TM antibody were noted using their labeling index as a percentage (%) in each specimen, and the measurements were averaged. The scores were as follows: score 0, no positive staining; score +, from 1% to 10%; score ++, from 11% to 50%; and score +++, more than 50% positive cells. Scores of ++ and +++ were classified as positive immunostaining, and scores of 0 and + were classified as negative immunostaining. The scoring standard is described in **S**1 **Fig**

### Cell cultures

The colon cancer cell lines (DLD-1 and HT-29 cells) were purchased from American Type Culture Collection (ATCC) and cultured in RPMI 1640 medium (Life Technologies, Grand Island, NY) containing 10% (v/v) fetal calf serum (FCS) in a humidified incubator at 37°C in 5% CO_2_.

#### The manipulation of TM expression in a colon cancer cell line

TM expression was silenced using MISSION shRNA clones (Sigma Chemical Co., St. Louis, MO), and stable selection based on puromycin resistance was performed as previously described [18,21]. The target mRNA sequence for the human TM gene (NM_000361) was 5’-cttgctcataggcatctccatc-3’. The MISSION non-targeted shRNA control vector (SHC002) sequence 5’-caacaagatgaagagcaccaa-3’ was used as a scrambled control. The overexpression plasmid pCDNA3-topo-TM was constructed as previously described [18]. The plasmids were transfected into the cells using a neon pipette-type microporator (Invitrogen Life Technologies, Grand Island, NY) as previously described [18,21,22]. TM expression levels were confirmed via Western blot analysis.

#### Western blot analysis

The samples were lysed using CelLytic M Cell Lysis Reagent (Sigma Chemical Co., St. Louis, MO) in the presence of a protease inhibitor (SERVA Electrophoresis GmbH, Heidelberg, Germany). The cell lysates were separated via 10% SDS-PAGE and electro-transferred to PVDF membranes (GE HealthCare, Piscataway, NJ). These membranes were subsequently incubated with anti-TM, anti-fibronectin, anti-ezrin, anti-vimentin, and anti-GAPDH antibodies (Santa Cruz Biotechnology, Inc., Santa Cruz, CA). Then, horseradish peroxidase-conjugated secondary antibodies (1:5000) were applied. Next, the protein bands were visualized using an enhanced chemiluminescence reagent (GE HealthCare, Piscataway, NJ) and detected using a VersaDoc 5000 imager (Bio-Rad Laboratories, Hercules, CA) [23–25].

#### Cell proliferation assays using the xCELLigence biosensor system

These experiments were modified from a previous study [26] and performed using an RTCA DP instrument (ACEA Biosciences Inc., San Diego, CA). Growth curves were constructed using 16-well plates (E-plate 16, ACEA Biosciences Inc.). We seeded 5,000 cells/well on an E-plate 16 in an FCS-containing medium; the plate was monitored once every 30 s for 4 h and then once every 30 min. The data analysis was conducted using RTCA version 1.2.

#### Cell migratory capacity determination

An in vitro cell migration assay was performed using an 8-μm BD Falcon^TM^ cell culture insert (BD Biosciences, San Jose, CA) [26]. Aliquots of 1 × 10^5^ cells were seeded on the upper compartment of the chamber; the lower compartment was filled with DMEM containing 10% FCS. After the cells were incubated for 24 h, the non-migrating cells that were on the upper surface of the membrane were removed. Then, the migrated cells were stained and counted under a microscope (Olympus IX71, Japan).

#### Cell migration assays using the xCELLigence biosensor system

These experiments were conducted using an RTCA DP instrument (ACEA Biosciences, Inc.) placed in a humidified incubator containing 5% CO_2_ and maintained at 37°C as previously described [24,26]. The cells (20,000 cells/well) were seeded on the upper chamber of a CIM-plate 16 in a serum-free medium for the migration assay. The data were analyzed using RTCA version 1.2.

### Wound healing assay

Cells were seeded on ibidi cell culture inserts (ibidi GmbH, Inc., Munchen, Germany) in 35-mm dishes and incubated at 37°C in 5% CO_2_. After 24 h, the culture inserts were removed and medium was added. Images of the cells were captured using a time-lapse microscope (Lumascope Model 500X video microscopy). The gap was analyzed using ImageJ software.

### Statistical analyses

All experiments were repeated at least three times. All data obtained from the cell proliferation and migration assays are expressed as the means ± standard deviations. The data presented in some figures are from single, representative experiments. Statistical significance was determined using Student's t test (two-tailed) or the chi-square test to compare two groups of data sets. All statistical calculations were performed using SPSS statistics 17.0 software (SPSS Inc. Chicago, IL, USA).

## Results

### TM expression levels correlated with the CRC clinical outcomes

**Fig 1:**
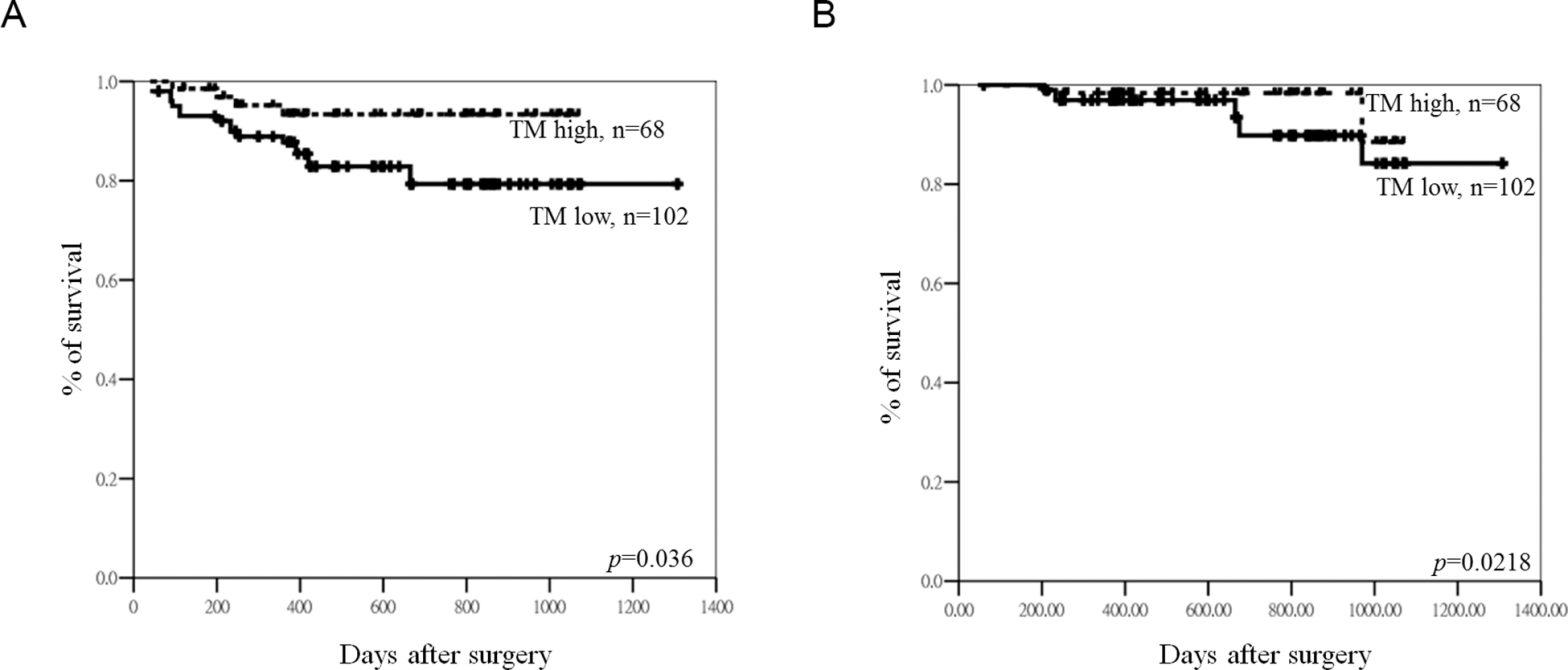
TM expression levels in tumor tissues from patients with CRC correlate with OS and DFS. Patients displaying lower TM levels exhibited reductions in both DFS (A) and OS rates (B). According to Kaplan-Meier analysis, the OS rate of patients displaying lower TM levels was lower than that of patients displaying higher TM levels (p = 0.0218). Low TM expression in patients with CRC was associated with a reduced DFS rate (p = 0.036).

To investigate the clinical role of TM in CRC, we examined the TM expression levels of the CRC specimens via IHC. The relationships between TM expression level and the CRC clinical outcomes were analyzed. As Fig 1 shows, the patients who displayed lower TM levels exhibited a lower disease-free survival (DFS) rate than those displaying higher TM levels according to Kaplan-Meier analysis (p = 0.036; Fig 1A). We also analyzed the association between TM expression and the overall survival (OS) rate of the patients. As Fig 1B shows, low TM expression was associated with a poor OS rate among patients with CRC (p = 0.0218). Because the clinical outcomes of the patients with CRC correlated with the depth of tumor invasion, the lymph node metastasis status and the pathological stage, we investigated whether TM expression correlated with these clinical parameters. In total, 170 tumor tissues were collected from patients with stage I to stage III CRC. As Table 1 shows, the level of TM expression did not significantly differ according to the pathological stage, the status of lymph node metastasis or the depth of tumor invasion; however, TM expression did differ by age. Furthermore, logistic regression analysis showed that tumor stage and TM expression acted as significant and independent recurrent risk factors (Table 2; p = 0.012, 95% CI = 1.335–10.100 for tumor stage; p = 0.028, 95% CI = 0.084–0.867 for TM expression). Patients without TM protein expression had a 3.714-fold higher risk of having CRC than those with TM expression. These results suggest that a lack of TM expression and late disease stage are equally significant, unfavorable and recurrent factors in CRC. These results suggest that TM expression influences the survival of patients with non-metastatic CRC.

**Table 1:** The associations between TM expression and clinical parameters in the tumor tissues of patients with CRC.

	TM		
Parameters	Low	High	p-value
	(n = 102)	(n = 68)	
Age (years)			
≤ 65	55	25	
> 65	47	43	0.030
Gender			
Male	42	28	
Female	60	40	1.000
T factor			
1	4	6	
2	23	11	
3	63	47	
4	12	14	0.227
N factor			
0	55	41	
1 + 2	47	27	0.412
Stage			
I	21	11	
II	33	29	
III	48	28	0.382

**Table 2:** Logistic regression analysis of the various potential recurrent factors in patients with CRC with different TM protein expression levels.

Variable	RR	Unfavorable/favorable	95% CI	p-value
TM protein	3.714	Negative/positive	0.084–0.867	0.028
Tumor stage	3.676	II/I	1.335–10.100	0.012
Gender	0.785	Male/female	0.305–2.204	0.219
Age	1.848	> 65/ ≤ 65	0.694–4.926	0.644

Logistic regression analysis was used for the statistical analysis.

### The effects of TM depletion on colon cancer cell proliferation and migration

**Fig 2:**
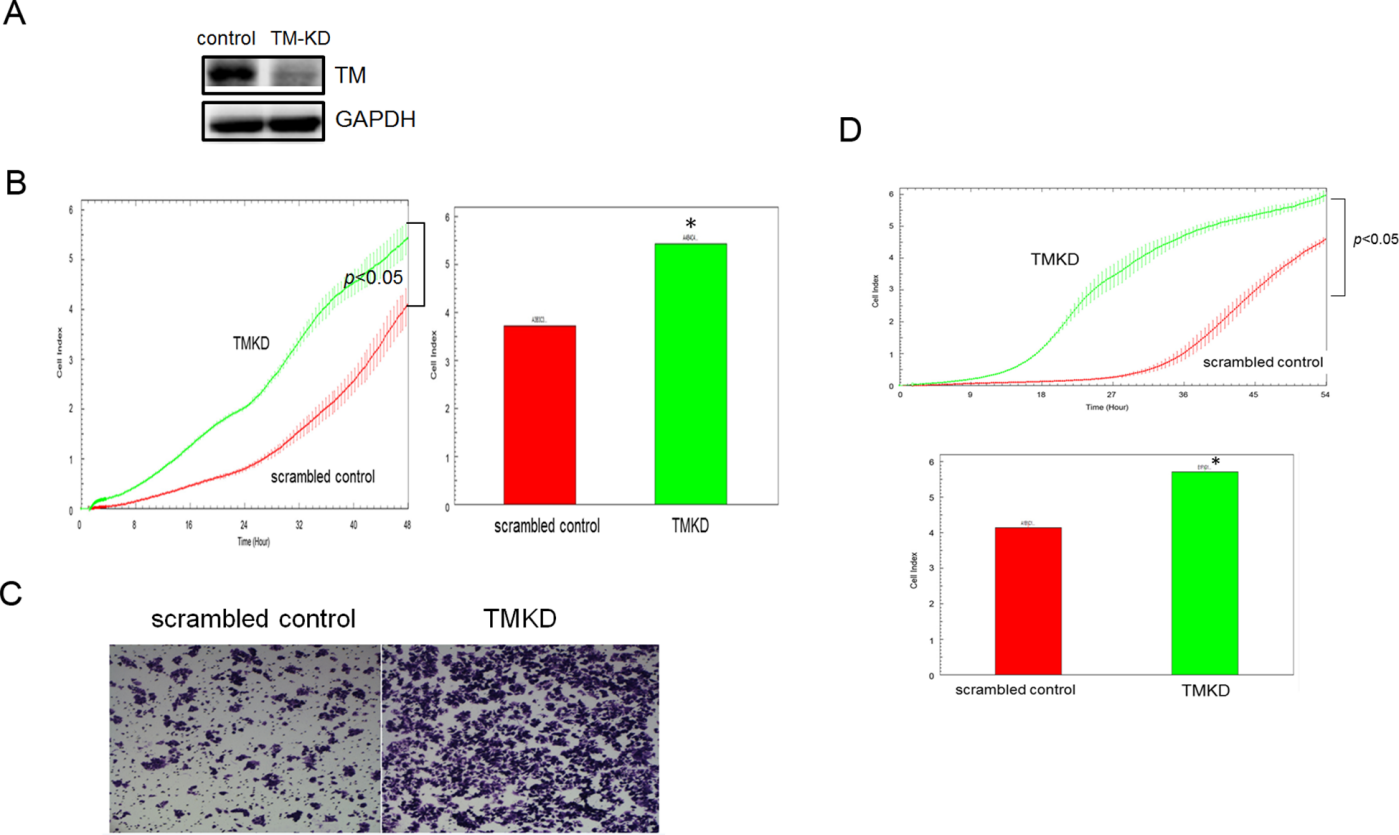
The effect of TM silencing on the proliferation and migration of DLD-1 cells. (A) TM-KD cells were generated using siRNA. Stably transfected cells were selected using antibiotics. TM expression levels were determined via Western blot analysis. GAPDH was used as a loading control. (B) The proliferative capacities of the scrambled control and TM-KD DLD-1 cells were determined using the xCELLigence biosensor system. The experiments were repeated at least three times independently in quadruplicate for each sample. (C-D) The migratory capacities of the TM-KD and scrambled control DLD-1 cells were determined using Transwell migration assays and the xCELLigence biosensor system. (C) In the Transwell migration assay, cells was harvested after 24 h of incubation. The experiment was repeated at least three times independently in duplicate each time. (D) In the biosensor system, the x-axis represents time (h), and the y-axis represents the cell index. The experiments were repeated at least three times independently in quadruplicate for each sample.

To detect endogenous TM expression in human colon cancer cells, the levels of TM in DLD-1 and Ht-29 cells were assessed via Western blot analysis. TM was highly expressed in DLD-1 cells but not in Ht-29 cells (data not shown). To determine the role of TM in colon cancer progression, we generated TM-knockdown (TM-KD) and scrambled control cells using shRNA. Stably transfected TM-KD and scrambled control DLD-1 cells were generated, and the TM expression level was determined (Fig 2A). We found that the proliferative ability of the TM-KD cells was lower than that of the scrambled control cells based on the results from the xCELLigence biosensor system (Fig 2B). Transwell migration assays revealed that the TM-KD DLD-1 cells exhibited a significantly increased migratory capacity compared with the scrambled control cells (Fig 2C). We also confirmed the migratory capacity of the stable cell lines using the xCELLigence biosensor system and found that TM-KD promoted DLD-1 cell migration (Fig 2D). The results indicated that TM silencing increased the proliferation and migration of colon cancer cells.

### The effects of TM overexpression on colon cancer cell migration

**Fig 3:**
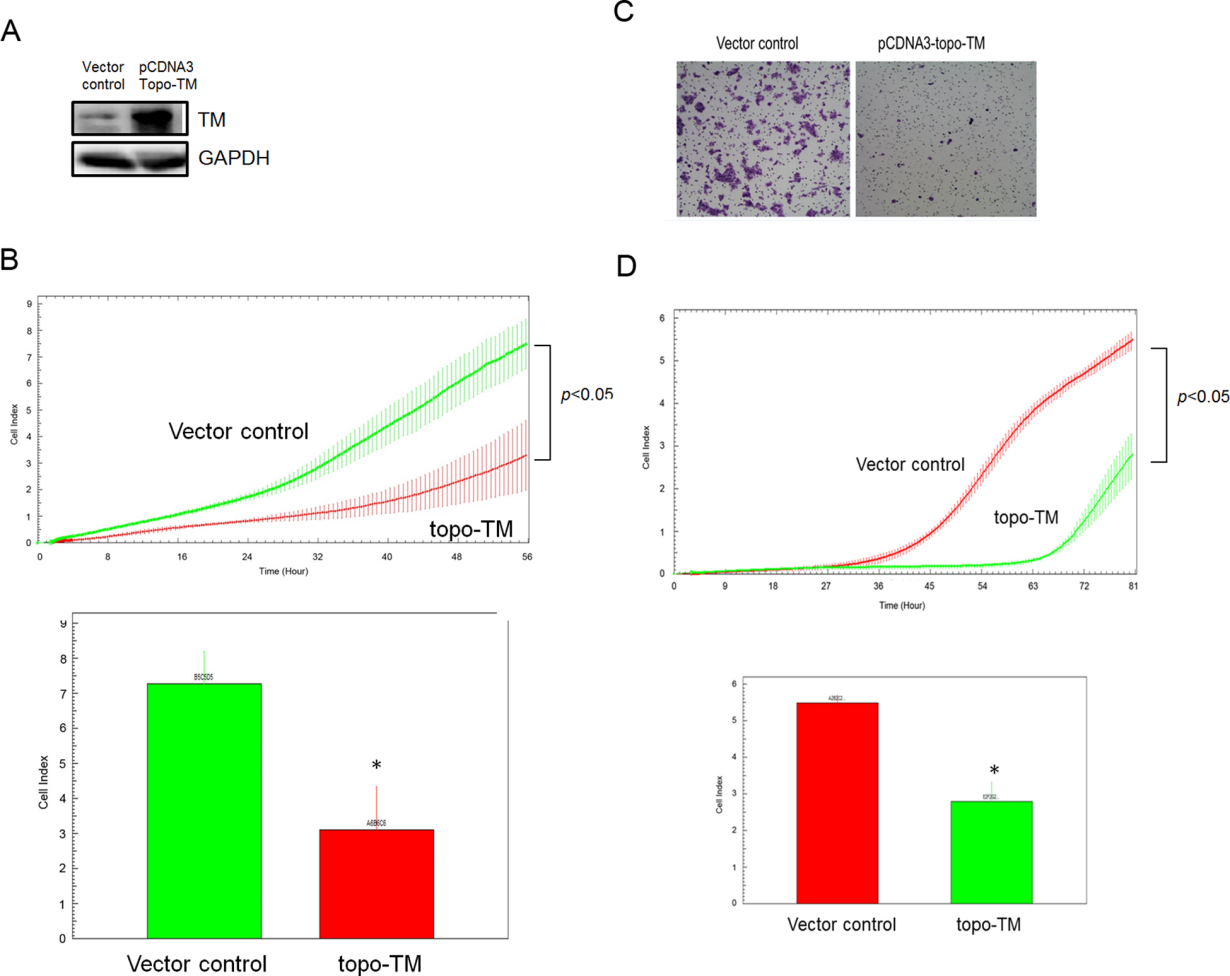
TM overexpression reduced cell proliferation and migration. (A) DLD-1 cells were transfected with pcDNA3-topo-TM or vector control plasmid. Stably transfected cells were selected using antibiotics. The expression levels of TM were analyzed via Western blot analysis. GAPDH was used as a loading control. (B) The proliferative capacities of the vector control- and pcDNA3-topo-TM-transfected cells were determined using the xCELLigence biosensor system. The experiments were repeated at least three times independently in quadruplicate for each sample. (C-D) The migratory capacities of the vector control- and pcDNA3-topo-TM-transfected cells were detected using the Transwell migration assay and xCELLigence biosensor system. (C) In the Transwell migration assay, cells were harvested after 24 h of incubation. The experiment was repeated at least three times independently in duplicate each time. (D) In the biosensor system, the x-axis represents time (h), and the y-axis represents the cell index. The experiments were repeated at least three times independently in quadruplicate for each sample.

To better understand the function of TM in colon cancer cells, we transfected the pcDNA3-topo-TM or vector control plasmid into DLD-1 cells. Elevated TM levels were confirmed via Western blot analysis (Fig 3A). Furthermore, based on the results from the xCELLigence biosensor system (Fig 3B), the proliferative capacity of DLD-1 cells was reduced when TM was overexpressed. Moreover, TM-overexpressing cells exhibited significantly decreased migration compared with the control vector-transfected cells (Fig 3C and 3D). We also confirmed that TM overexpression in HT-29 cells inhibited their migratory ability (S2 Fig). These results indicate that TM overexpression reduces the proliferative and migratory capacities of colon cancer cells.

### TM mediates cell proliferation and migration via the EMT biomarker cyclooxygenase (COX)-2

**Fig 4:**
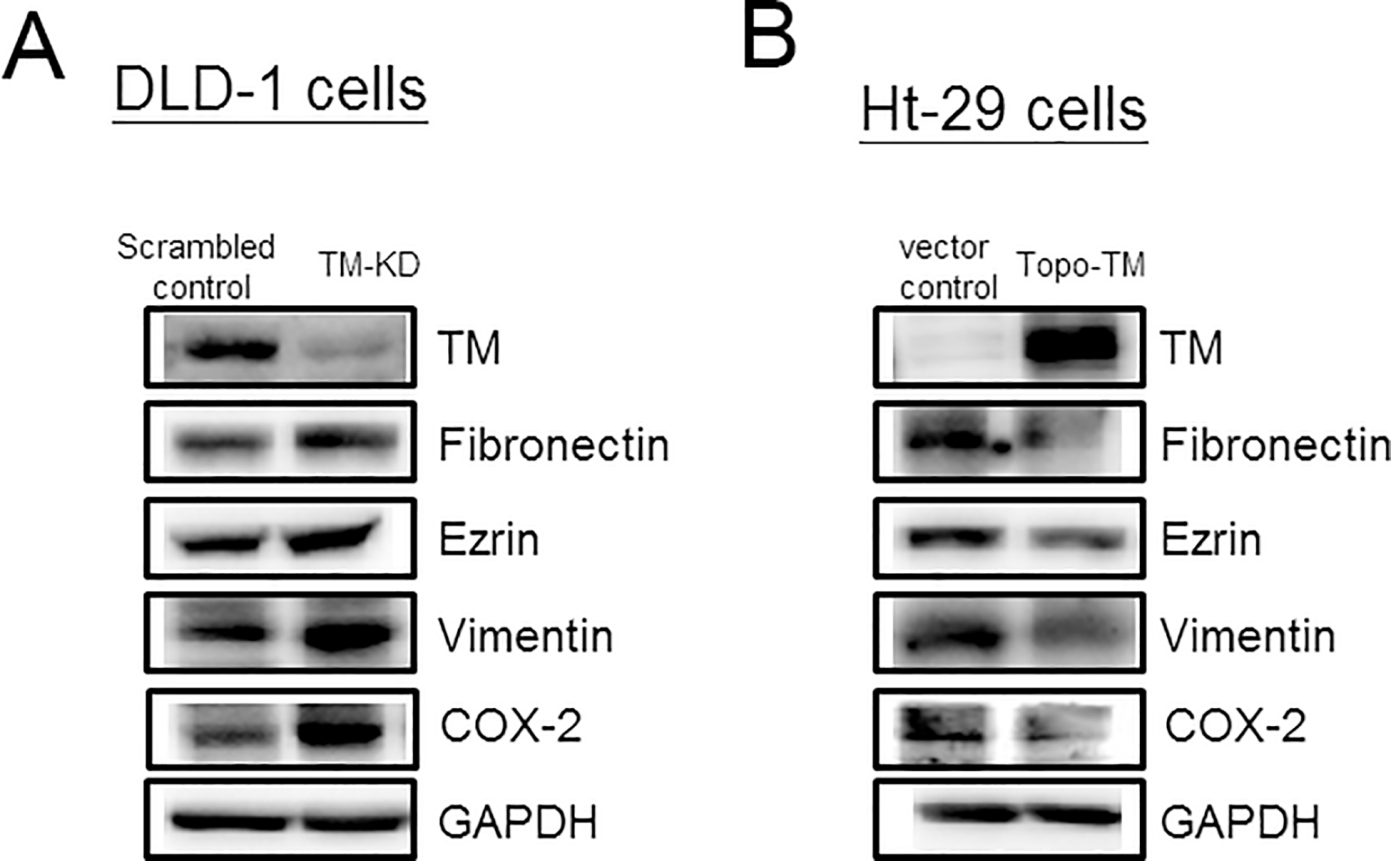
TM negatively regulates the expression of EMT markers in CRC cells. (A) The expression levels of epithelial and mesenchymal markers (fibronectin, ezrin, and vimentin) as well as COX-2 were analyzed in scrambled control and TM-KD DLD-1 cells via Western blot analysis. GAPDH was used as a loading control. (B) The expression levels of epithelial and mesenchymal markers as well as COX-2 were compared between the vector control- and pcDNA3-topo-TM-transfected HT-29 cells via Western blot analysis. GAPDH was used as a loading control. Mesenchymal markers were up-regulated in TM-KD cells but down-regulated in pcDNA3-topo-TM-transfected cells compared with the corresponding control cells.

Finally, we determined the mechanism that underlies the migratory responses regulated by TM by evaluating the levels of EMT markers such as fibronectin, ezrin, and vimentin. After the successful suppression of the TM levels in DLD-1 cells, fibronectin, ezrin, and vimentin expression was markedly up-regulated (Fig 4A). The expression levels of these mesenchymal markers were reduced in the stably pcDNA3-topo-TM-transfected cell line compared with the control Ht-29 cell line (Fig 4B). In addition, we found that COX-2 expression was increased in the TM-KD cells compared with the scrambled control cells. Together, our findings indicate that TM is directly involved in the migration of colon cancer cells by modulating the expression of EMT markers.

## Discussion

The current study attempted to identify the TM expression pattern of Asian patients with CRC and the TM mechanism that influences CRC survival. In this study, 40% of CRC specimens showed high TM expression; Hanly et al. reported that 26.3% of tumors expressed TM and that 73.5% of tumors showed no neoplastic cell staining [16]. However, the positive rate in our study was higher than that reported in the study by Hanly and colleagues. If the definition of positive immunostaining used by Hanly et al. is modified, then the positive percentage is higher. In addition, TM expression was independent from the depth of tumor invasion and the nodal status in this study. According to the results, the expression of TM in the CRC specimens might differ among ethnicities. This study demonstrated that patients displaying low TM expression in their CRC cells also exhibited a higher risk of recurrence. Low TM expression causes increased expression of COX-2, which induces EMT by increasing fibronectin and vimentin levels. In addition, the expression levels of ezrin are increased in colon cancer cells displaying low TM expression.

Metastasis is the predominant cause of death among patients with CRC. The process of metastasis likely involves the migration of individual cancer cells that detach from a primary site and travel through the circulation to distant organs [3]. The detachment of cancer cells is partially characterized by the ability of a cancer cell to overcome cell-cell adhesion and invade the surrounding tissue; this underlying process is known as EMT [27].

Fibronectin, vimentin, and ezrin are regarded as important hallmarks of EMT [28–31]. Fibronectin is expressed in CRC tissues and mediates the migration of CRC cells in vitro [28–30]. As an important component of the tumor microenvironment, the alternative extra domain A of fibronectin promotes the vasculogenesis, tumorigenesis and metastasis of colorectal tumors by inducing EMT [32]. Vimentin is an intermediate filament protein, and the expression of vimentin is thought to be a prerequisite for EMT induction [33]. Previous studies have demonstrated that vimentin plays a role in the regulation of focal adhesions [34,35]. Ezrin belongs to the ezrin-radixin-moesin (ERM) family [36]. Ezrin is expressed at higher concentrations in the microvilli and in the ruffling membranes of various cell types, and it is a component of the intestinal brush border microvilli [36,37]. Previous studies have demonstrated that ezrin plays important roles in cell motility, invasion and distant metastasis [38–40]. In addition, TM was identified as an ezrin-interacting protein [41]. In this study, TM suppression in colon cancer cells enhanced fibronectin, vimentin, and ezrin expression levels, which could increase the migratory capacity of colon cancer cells.

COX-2 was also reported to increase the motility of colon cancer cells via EMT [42]. COX, an enzyme in the prostanoid biosynthetic pathway, has received attention due to its role in human cancers. COX-2, an inducible COX isoform, is frequently overexpressed in patients with CRC, and CRC cases displaying high COX-2 expression are associated with a poor prognosis [43]. Furthermore, COX-2 was shown to regulate the expression of fibronectin and vimentin [44–46]. In this study, the suppression of TM was associated with increased expression of COX-2, which could induce the up-regulation of fibronectin and vimentin.

## Conclusions

Our data provide evidence that TM influences the survival of patients with non-metastatic CRC through EMT. Patients displaying low TM expression in their cancer cells exhibited reduced OS and DFS rates after curative surgery. Possible mechanisms underlying this effect include the increased expression of COX-2, EMT markers (fibronectin and vimentin) and an ERM protein (ezrin) in CRC cells displaying low TM expression.

## Supporting Information

S1 Fig TM expression in normal colonic mucosa and, primary colonic adenocarcinoma. (A-B) The TM expression patternprofile of in the normal colonic epithelium. (C-D) Low TM expression in primary colonic adenocarcinoma. (E-F) High TM expression in primary colonic adenocarcinoma. (Original magnification: A, C, and E: low- power field = 100X; B,D, and F: high- power field = 200X).
(PPTX)

S2 Fig TM overexpression suppressed the migratory ability in HT-29 cells. 5 x 105 HT-29 control and pCDNA3-topoTM HT-29 cells were seeded into ibidi cell culture inserts. After 24h, the culture-inserts were removed and added to the media. Images of the cells were captured using a time-lapse microscope.
(PPTX)
