# Supplementary figures and images for: Thrombomodulin Influences the Survival of Patients with Non-Metastatic Colorectal Cancer through Epithelial-To-Mesenchymal Transition (EMT)
Source: PLoS One. 2016 Aug 11;11(8):e0160550. doi: 10.1371/journal.pone.0160550 (PMC4981396; doi:10.1371/journal.pone.0160550)

## Slide 1
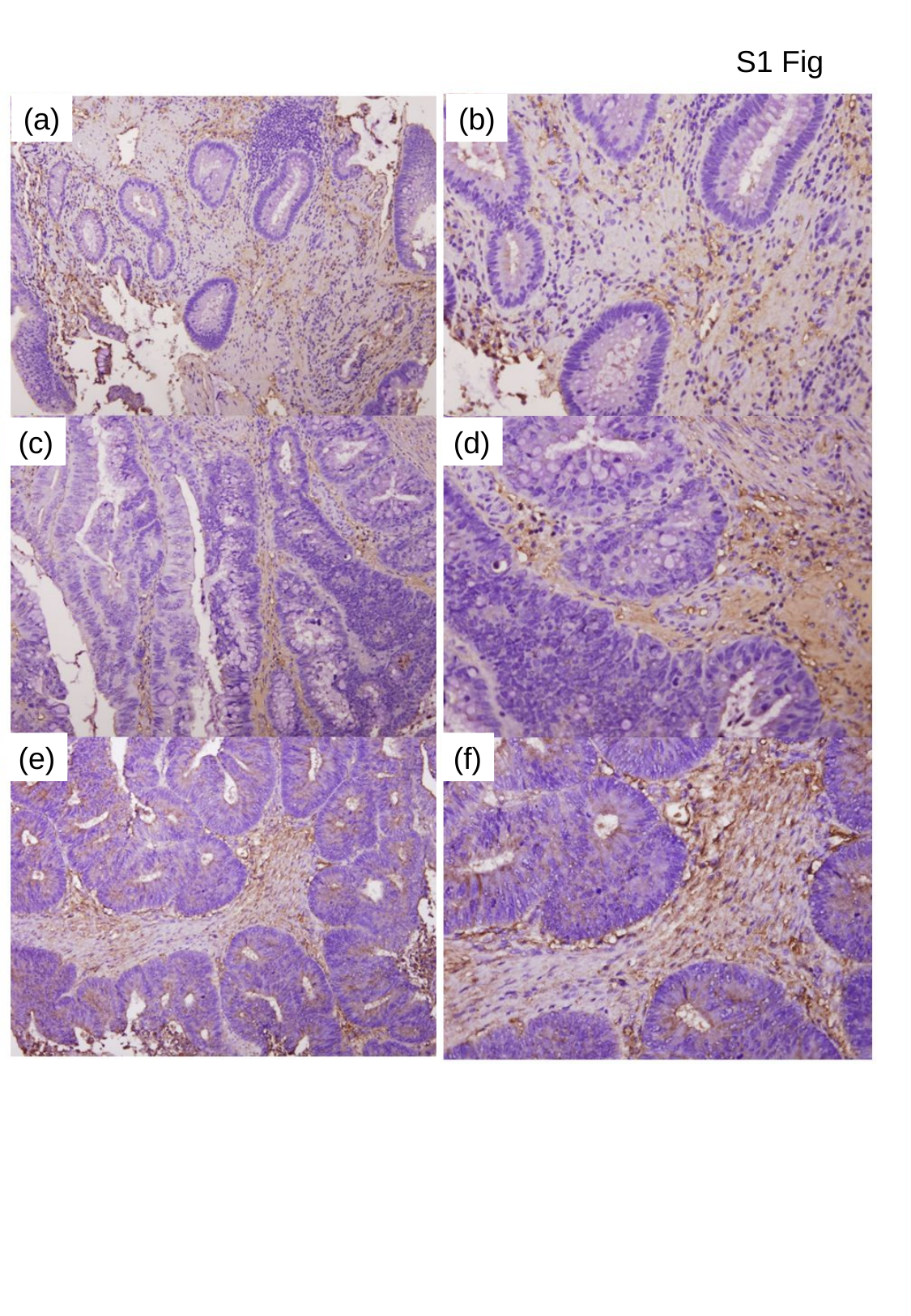

S1 Fig
(a)
(b)
(c)
(d)
(e)
(f)

Supplement: S1 Fig — (A-B) The TM expression patternprofile of in the normal colonic epithelium. (C-D) Low TM expression in primary colonic adenocarcinoma. (E-F) High TM expression in primary colonic adenocarcinoma. (Original magnification: A, C, and E: low- power field = 100X; B,D, and F: high- power field = 200X). (PPTX) [file pone.0160550.s001.pptx]

## Slide 1
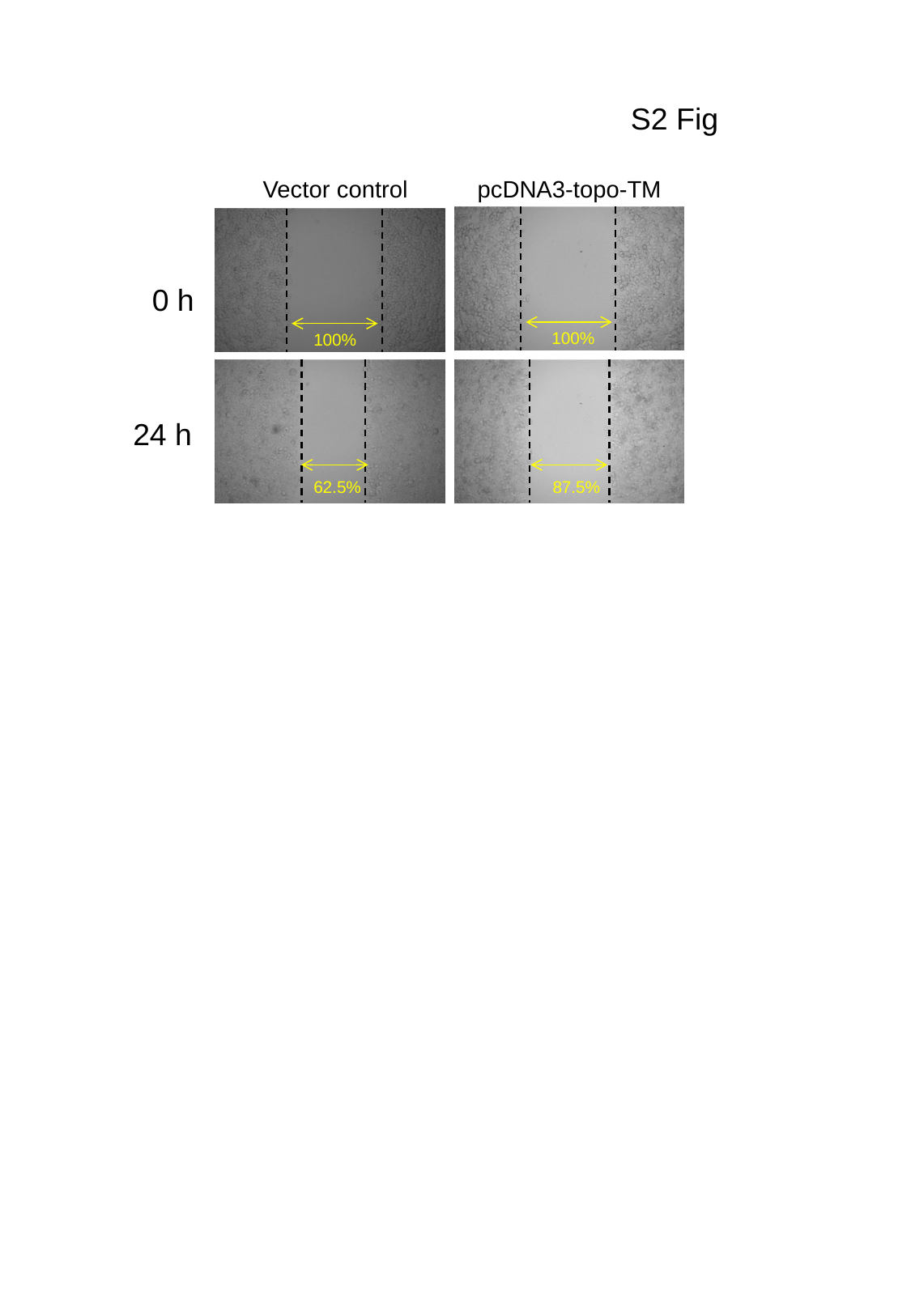

S2 Fig
Vector control
pcDNA3-topo-TM
100%
100%
62.5%
87.5%
0 h
24 h

Supplement: S2 Fig — 5 x 10^5^ HT-29 control and pCDNA3-topoTM HT-29 cells were seeded into ibidi cell culture inserts. After 24h, the culture-inserts were removed and added to the media. Images of the cells were captured using a time-lapse microscope. (PPTX) [file pone.0160550.s002.pptx]
